# Hematometra Caused by Chronic Lichen Planus in a Patient Mimicking Acute Urinary Retention: A Case Report

**DOI:** 10.7759/cureus.44736

**Published:** 2023-09-05

**Authors:** Munir Al-Ghazawi, Mohammed Saad, Hamza Salameh

**Affiliations:** 1 Urology, Barts Health National Health Service (NHS) Trust, London, GBR; 2 Orthopedics, North Devon District Hospital, Barnstaple, GBR

**Keywords:** bladder scan, lichen planus, hematometra, acute urinary retention, urology

## Abstract

The association between lichen planus (LP) with subsequent hematometra and acute urinary retention (AUR) is quite rare, and few cases reported that association. In such cases, if the patient is left untreated, then possible kidney injury and bladder wall rupture may arise from that retention. In this case, we reported a patient who is known to have a background history of long-standing LP that presented with a history and features suggestive of AUR and was found to be caused by hematometra on an ultrasound scan.

## Introduction

Acute urinary retention (AUR) is a condition that is characterized by a painful inability to pass urine from the urinary bladder outside the body [1], and this is usually caused by either obstruction of the urine outflow or neuromuscular failure of the bladder muscles. Leaving this condition without intervention may cause harm to the patient, starting from kidney injury and possible bladder wall rupture and subsequent urine leak.

On the one hand, hematometra is a condition that is described as the presence of blood inside the uterus and is usually caused due to mechanical etiology such as a vaginal septum or imperforate hymen [2]. This condition is usually spotted by a history of amenorrhea (primary or secondary) and abdominal mass on an exam. However, the gold-standard test is ultrasound to diagnose it.

On the other hand, lichen planus (LP) is an erosive condition that affects the skin and the genitalia and might cause a labia minora fusion if it is long-standing [3]. This fusion might cause a mechanical obstruction to the vaginal secretions as well as the menstrual blood and might cause a retention of that blood in the uterus.

## Case presentation

A 53-year-old female patient presented to the ED with an 11-hour history of lower abdominal pain and inability to pass urine. The patient denied any other symptoms during or prior to this presentation. Her medical background history showed that she is known to be hypertensive and has had LP for more than 10 years which is managed by only creams (once needed) that were prescribed by her general practitioner. In addition, the patient recalled that she stopped having her menses for the last 11 months.

On physical examination, her vitals were all within normal range, and upon general inspection, there were no obvious skin lesions or old scars related to her LP; however, only a very few old white patches on the buccal mucosa were noted. On the other hand, there was a suprapubic tenderness noted upon abdominal examination. The bladder edge was not appreciated, and no mass was felt during that exam. Her vaginal examination showed a fused labia minora without any skin lesions.

Basic blood was performed, including venous blood gas, full blood count, and urea + electrolytes, and all results were within normal and acceptable ranges. Subsequently, a bedside bladder scan was performed and showed a bladder volume of 520 mls. Diagnosis of AUR was made, and the decision to insert a urinary catheter to resolve her retention was made by an ED doctor.

Initially, the urinary catheter insertion was attempted by a healthcare assistant, who noted that the inner labia (labia minora) was so small and tight that she had to try multiple times in order to insert the catheter without getting any urine back. Another attempt was made by an ED doctor, who had the same difficulty and the same outcome.

Based on that, a referral was made to the on-call urology doctor in order to assess this patient. The same history was obtained and the fused labia minora was noted. Subsequently, a 14 Fr (French) two-way Foley was inserted smoothly, and the very minimal amount of clear yellow urine was drained; therefore, the position of the catheter was confirmed to be within the urinary bladder, and a repeat bladder scan each hour for the next two hours was ordered. The two repeated bladder scans showed the same amount of 520-525 mls each time. Hence, another possible differential diagnosis was raised.

**Figure 1:**
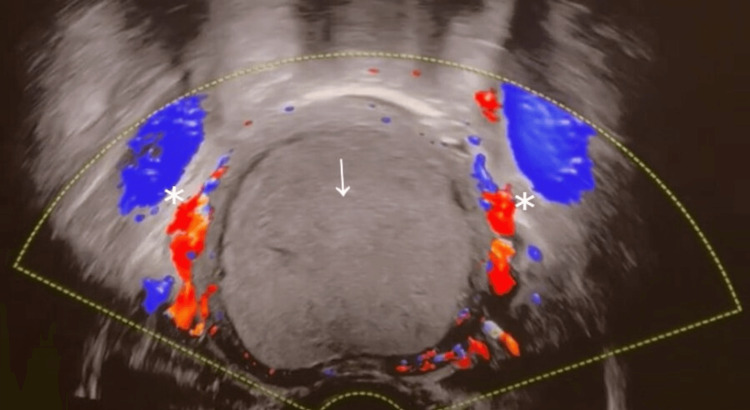
Hematometra on ultrasound scan (arrow) Hematometra seen as homogeneous fluid collection on the ultrasound scan (marked by an arrow). In addition, you can clearly note the normal blood supply around the uterus which has no continuation with the hematometra itself (both stars).

The urology team contacted the gynecology on-call doctor, who advised performing a trans-abdominal ultrasound scan to rule out any ovarian/endometrial pathology that might cause this presentation. An urgent ultrasound scan was performed and showed a 10.1 x 7.2 x 6.9 cm (500 mls) homogenous fluid collection within the uterus which is highly suggestive of hematometra (Figure 1).

The patient was transferred to the women's health department at the same hospital to be evaluated pre-operatively and was booked for dilation and curettage along with evacuation of the above-mentioned hematometra. The patient went home on the second day post-operatively without any complications recorded. Ultrasound of the urinary tract was performed after the surgery and was unremarkable. Moreover, the patient had her menses back after the surgery.

## Discussion

LP is a form of dermatosis that is usually missed when it affects the genitalia, unlike the classical variant, and the vulvovaginal form is a prime example of that. Moreover, the scarring formed can cause a decrease in quality of life [4]. This scarring might lead to the fusion of the labia minora, forming a degree of mechanical obstruction to the vaginal secretions including menstrual blood and urinary outflow. Essentially, UTIs and hematometra may develop. In a study by Helgesen et al., which had just under 1,000 female patients, the median age at which symptoms started for erosive LP was about 51 [5]. This age is very close to the average age of menopause, and this could be a reason why our patient didn't seek medical attention at that time, despite the fact she had the LP for the last 10 years prior to presentation.

The majority of the reported cases of AUR due to an external gynecological compression were due to mechanical obstruction of menstrual blood by the imperforate hymen that led to either hematocolpos (blood in the vagina) or hematocolpometra (blood in the vagina and uterus) that eventually led to acute retention [6-8].

It is worth mentioning that we performed a trans-abdominal ultrasound scan rather than a trans-vaginal one due to the tight and fused labia minora in order to prevent the patient from any pain or discomfort. Another fact to mention is that the initial bladder scan performed failed to differentiate between urine (which appears black on ultrasound) and hematometra (which appears a shade of grey and more echogenic), and this is considered a limitation of ultrasound in the differential diagnosis.

Although our patient didn't present with a true AUR due to the fact that her bladder was compressed by that hematometra, the patient still had some overlapping lower UTIs with lower abdominal pain that indicated a urinary catheter insertion. Alternatively, in cases where there is no significant post-void residual in the setting of an external compression of the urinary bladder, a careful assessment of the upper urinary tract with an ultrasound is a must, in order to rule out any hydroureteronephrosis and associated kidney injury, and that the reason why we performed that scan for this patient before her discharge.

## Conclusions

This patient presented with AUR that resulted from external compression of the bladder by a hematometra, and the initial assessment and management performed by the ED team was very reasonable; however, the inability to drain any urine back despite the correct placement of the urinary catheter should warrant an urgent urology referral in such presentation. Ultimately, repeated bedside bladder scans with almost the exact same readings and confirmed placement of the catheter within the bladder made us consider another differential diagnosis, which was hematometra managed by gynecology.

Even though the combination of LP and subsequent hematometra is rare, and the association between that hematometra and the resultant AUR is even more uncommon, the index of suspicion should be high enough in order to spot that diagnosis.

Furthermore, keep in mind that the local compression of that mass might mimic the AUR signs and symptoms or even cause one. Essentially, repeated bedside bladder scan readings with confirmed intravesical urinary catheters should be enough reasons to raise the urgency to perform a formal ultrasound scan in order to rule out any potential pathology.
